# Think twice before you tell

**DOI:** 10.1007/s12471-014-0535-y

**Published:** 2014-02-21

**Authors:** A. A. M. Wilde

**Affiliations:** Academic Medical Centre Amsterdam, Amsterdam, the Netherlands

## Rhythm puzzle - question

A 34-year-old female comes to your outpatient clinic because of her family history. Her father died suddenly at age 42, without prior complaints and her brother died suddenly at age 35. In the years before, he was known with shortness of breath and signs of a cardiomyopathy (LVEF 40 %).

**Fig. 1:**
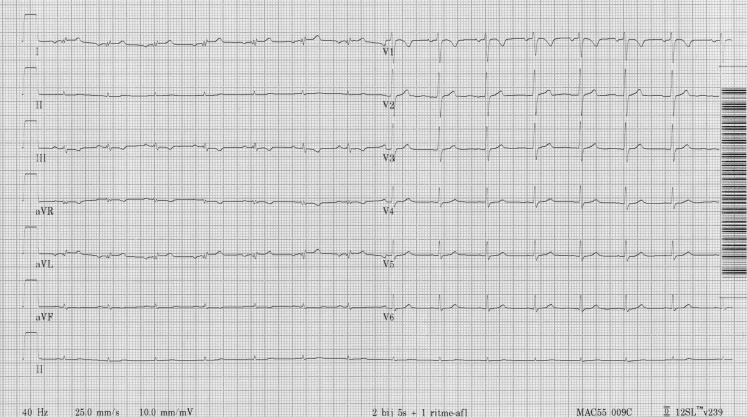
ECG at presentation

She herself has no symptoms whatsoever. She plays active volleyball. On physical examination you do not find any abnormalities and her echo is completely normal. The ECG is shown in Fig. 1. What would your diagnosis be? Are you worried and/or should she be worried?

The answer to this Rhythm Puzzle can be found elsewhere in this number.

